# *Lycium barbarum* polysaccharides alleviate cadmium-induced renal injury in ducks by activating the Nrf2-mediated antioxidant defense pathway

**DOI:** 10.3389/fvets.2026.1998071

**Published:** 2026-10-14

**Authors:** Xueru Wang, Dongdi Liu, Yonggang Ma, Hui Zou, Xishuai Tong, Jiaqiao Zhu, Ruilong Song, Hongyan Zhao, Zongping Liu

**Affiliations:** 1 College of Veterinary Medicine, Yangzhou University, Yangzhou, Jiangsu, China; 2 Institute of Agricultural Science and Technology Development (Joint International Research Laboratory of Agriculture and Agri-Product Safety of the Ministry of Education of China), Yangzhou University, Yangzhou, Jiangsu, China; 3 Jiangsu Co-Innovation Center for Prevention and Control of Important Animal Infectious Diseases and Zoonoses, Yangzhou, Jiangsu, China; 4 College of Animal Science and Technology, Xinyang Agriculture and Forestry University, Xinyang, Henan, China

**Keywords:** antioxidant defense pathway, cadmium, duck, *Lycium barbarum* polysaccharides, Nrf2, renal injury

## Abstract

Cadmium (Cd) is a widespread environmental contaminant related to renal immunosuppression and metabolic dysfunction. *Lycium barbarum* polysaccharides (LBPs) are active extracts of wolfberry and possess a variety of excellent properties. However, the specific mechanism of action for Cd and LBP remain unclear. In the present study, *Shaoxing ducks* were used to establish an *in vivo* model to elucidate the effects of combined Cd and LBP exposure on the kidneys and the underlying mechanisms. The ducks were exposed to CdCl_2_ (50 mg/kg) and/or LBP (50 mg/kg) for 30 days. We observed that Cd exposure increased the kidney index and the levels of the renal function indicators CREA and UA. Further research revealed that Cd exposure caused histopathological damage and ultrastructural alterations. In addition, Cd accumulation induced oxidative stress by disrupting ionic homeostasis and inhibiting the Nrf2-mediated antioxidant defense pathway. Finally, we found that Cd promoted lipid accumulation, disrupted lipid metabolism, and thereby induced renal immunosuppression. Notably, LBP effectively alleviated Cd-induced renal injury, suggesting beneficial effects against Cd-triggered nephrotoxicity.

## 1 Introduction

Cadmium (Cd) is a toxic heavy metal pollutant that is widely distributed in nature. Due to its excellent corrosion resistance, it is widely used in industrial applications, such as electroplating, batteries, and pigments (1). However, with the rapid development of industrialization and the increase in the discharge of “three wastes,” Cd concentrations in water, soil, and air have also increased, posing a significant threat to human and animal health and the ecological environment. As early as the mid-twentieth century, “Itai-itai disease,” a disease characterized by severe pain, kidney dysfunction, and serious bone deformities, was confirmed to be directly related to Cd poisoning. At present, there is abundant evidence indicating that long-term exposure to Cd affects various systems, mainly including the respiratory, nervous, reproductive, and urinary systems (2). Among these, the kidney is the main site for Cd deposition and toxicity, absorbing approximately half of the total Cd in the metabolic process. The kidney is particularly sensitive to Cd stress. Under different exposure models and times, Cd induces nephrotoxicity through multiple molecular mechanisms, such as disruption of ion homeostasis, induction of oxidative damage and inflammation, and activation of apoptosis, pyroptosis, and autophagy, ultimately leading to pathological tissue damage and declines in renal function (3–5). Oxidative stress and ion homeostasis are two closely interwoven and mutually influencing core processes in cellular physiology. The relationship between them can be summarized as a vicious cycle of mutual dependence and mutual destruction. Therefore, Cd pollution and its toxic effects have become significant public health and environmental issues worldwide, and there is an urgent requirement for continuous attention and effective governance.

The kidney is not only an important excretory organ but also an active immune organ. The glomerulus is the renal core functional unit, which acts like a precise filter screen. When antibodies and antigens in the blood combine to form “immune complexes,” these complexes can flow through the glomerulus with the blood and deposit in different parts of the filter (subepithelial, subcutaneous, or mesangial areas), thereby activating the complement system and triggering a strong inflammatory response, leading to glomerular damage (6). However, the kidney is not simply a passive tissue susceptible to damage; it possesses abundant resident and migratory immune cells, which together form the renal immune microenvironment. Macrophages are the most abundant immune cells in the kidney. In a healthy state, they play a role in monitoring and eliminating foreign substances; in a diseased state, they become activated and release a large amount of inflammatory factors, thereby exacerbating tissue damage while also participating in subsequent tissue repair (7). T cells are the core cells of the adaptive immune system. When the kidney is exposed to stress, infection, or autoimmune reactions, T cells can become extensively activated and recruited to the kidney, transforming from “spectators” to “main participants.” They are one of the “key decision-makers” that determine the immune status and health of the kidney. The immune system of the organism is extremely sensitive to external pollutants and can resist the invasion of pathogens (8). Therefore, the immune system is often used to assess the potential risk of certain pollutants, which is vital for exploring their adverse effects. Xu et al. (9) revealed that Cd blocked the neural development of zebrafish and suppressed the neuroimmune response. Yin et al. (10) also reported that the abnormal behavior of snails, the destruction of cell structure, the decline in immune function, and the occurrence of apoptosis were related to Cd toxicity. Studies have confirmed that Cd suppresses immune function and alters inflammatory responses in the duck spleen, as evidenced by increased expression levels of interleukin 6 (IL-6), tumor necrosis factor-α (TNF-α), and interferon -γ (IFN-γ), together with decreased expression levels of interleukin 10 (IL-10) and interleukin-1β (IL-1β) (11). Inflammatory factors are small-molecule proteins secreted by immune cells, which are used for communication between cells. The immune system, especially under conditions of chronic inflammation, can profoundly alter lipid metabolism throughout the body.

Lipids are not only the basic structural components of cell membranes but also serve as crucial sources of energy storage and supply, playing vital roles in all living organisms. In simple terms, lipid metabolism can be understood as the entire process through which the body digests, absorbs, synthesizes, breaks down, and transports lipid substances. When kidney function is impaired, lipid metabolism disorder characterized by a significant increase in triglycerides (TGs) may occur. Non-targeted lipidomic analysis has disclosed that environmental Cd pollution alters cardiac lipid metabolism, increases the levels of pro-inflammatory lipids, and disrupts lipid distribution. Moreover, Cd influences the production of inflammatory factors by regulating the NF-κB signaling axis, causing inflammation in the mouse heart (12). Cd exposure aggravates the development of diabetes by promoting intracellular lipid accumulation, inhibiting lipid degradation, disrupting lipid metabolism, and impairing pancreatic β-cell function (13). Studies have found that high Cd concentrations disrupt the normal metabolic process of lipids in *Bufo gargarizans* by enhancing lipid synthesis, reducing fatty acid *β*-oxidation, altering thyroid hormone secretion, and inducing oxidative damage in the liver (14). In poultry, Cd exposure induces oxidative stress and inflammation and increases the levels of total cholesterol (TC) and TG in the kidney, thereby leading to lipid metabolism disorder (15). The relationship between the kidney and lipid metabolism is closely interconnected: “diseased kidney leads to lipid disorder, and lipid disorder impairs kidney function.” Controlling blood lipid levels is crucial for addressing this health issue. Although maintaining regular routines and implementing effective protective measures are important, medication treatment is often necessary. However, effective drugs with minimal side effects are still limited. Therefore, it is necessary to develop and screen for effective intervention drugs.

*Lycium barbarum* is a small red berry with extremely high nutritional value. It is usually used in daily cooking and is also a traditional Chinese medicine. *Lycium barbarum* polysaccharides (LBPs), the major bioactive components of *Lycium barbarum*, exhibit antioxidant and anti-inflammatory properties. Accumulating evidence indicates that LBPs confer protective effects against renal injury induced by various toxicants, partly by regulating oxidative stress and inflammatory responses. A previous study showed that LBPs increased the number of muscle satellite cells, significantly increased the muscle-to-body weight ratio, reduced fat content, and enhanced muscle endurance in adult mice (16). Interestingly, LBP combined with aerobic exercise can mitigate hepatic inflammation and lipid accumulation by restoring the alterations in intestinal microbiota composition and metabolism caused by a high-fat diet in rats (17). Accumulating evidence suggests that LBP exerts a wide range of biological effects, including anti-inflammatory, antioxidant, anti-aging, hypoglycemic, hypocholesterolemic, radioprotective, immunomodulatory, antitumor, and neuroprotective effects, through multiple signaling pathways (18). With advances in science and technology, the beneficial effects of LBP have been supported by both traditional experience and modern research. It can not only be part of a healthy human diet but also be widely used as a promising candidate feed additive in animal nutrition. For instance, dietary supplementation with LBP enhanced antioxidant defenses, immune function, and growth performance in broilers (19). In piglets, LBP reduced the abundance of Firmicutes in the intestinal tract and enhanced antioxidant capacity (20). LBP also shows potential as a therapeutic agent for hyperlipidemia, diabetes, liver disease, and other related disorders (21).

Ducks represent an important component of waterfowl production and provide high-quality protein for human consumption, especially in Jiangxi Province. As aquatic waterfowl, ducks inhabit ponds, lakes, and rivers. Occupying a relatively high trophic level in aquatic food chains, ducks are more susceptible to aquatic pollutants than many terrestrial species. Ducks possess considerable economic value: Duck meat and by-products such as offal enter the food supply chain. Therefore, exploring the mechanisms underlying Cd-induced renal injury in ducks and identifying effective feed additives are essential for safeguarding food safety and public health. The objective of this study was to explore the effects of Cd exposure on the kidney of waterfowl and the beneficial mechanisms of LBP, thereby deepening our understanding of the effects of heavy metal Cd pollution on waterfowl health.

## 2 Materials and methods

### 2.1 Ethics statement

Animal experiment protocol was accepted and approved by the Institutional Animal Care and Use Committee of Yangzhou University (Approval number: 202403056).

### 2.2 Experimental animals and treatment

In total, 40 healthy 7-day-old male *Shaoxing ducks* were purchased from a commercial hatchery and were housed in an environmentally controlled room under a 12 h light/12 h dark cycle. The room temperature and relative humidity were maintained at 28 ± 2 °C and 60 ± 5%, respectively. After adapting for a week, the ducks were randomly assigned to four groups (*n* = 10 per group): The control group, LBP group, Cd group, and Cd + LBP group. Cd was administered by mixing it with the feed (50 mg/kg), while LBP was administered intragastrically (50 mg/kg). Cd and LBP doses used in this study were selected according to previous relevant studies and our preliminary experiments (22). The experimental design is shown in Figure 1A. Cadmium chloride (CdCl_2_) and LBP were obtained from Sigma-Aldrich (202908-50G) and Yangling Ciyuan Biotechnology Co. Ltd. (Xi’an, China), respectively.

**Figure 1:**
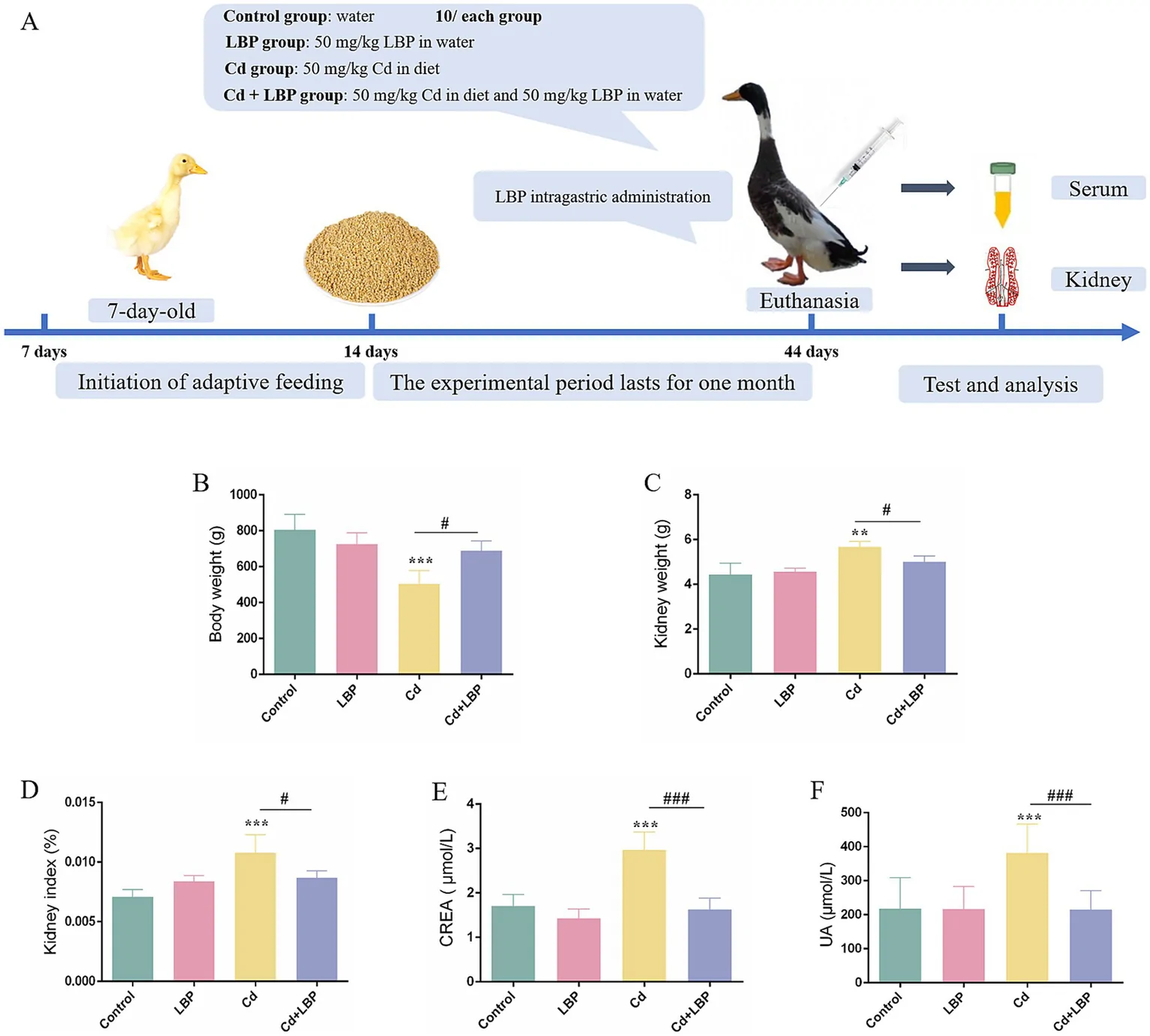
LBP restored renal ion homeostasis disrupted by Cd exposure. **(A)** Cd content. **(B)** Fe content. **(C)** Mn content. **(D)** Zn content. **(E)** Cu content. **(F)** Heatmap analysis. The heatmap color gradient shows row-wise Z-score-normalized element levels (positive = higher relative content and negative = lower relative content). Darker colors indicate lower element content, while lighter colors represent higher element content. Cd, cadmium; Fe, iron; Mn, manganese; Zn, zinc; and Cu, copper. All results are presented as the mean ± SD. *p < 0.05, **p < 0.01, and ***p < 0.001 compared to the control group; #*p* < 0.05, ##*p* < 0.01, and ###*p* < 0.001 compared to the Cd group; ns represents *p* > 0.05.

### 2.3 Sample collection

The experiment lasted for 30 d, and the body weight of the ducks was measured. After anesthesia, blood and kidney samples were quickly collected and preserved under appropriate conditions for subsequent trials.

### 2.4 Kidney index

The kidney index was calculated as follows: Organ index = Organ weight (g) / Body weight (g) × 100%. This ratio reflects the relative relationship between the kidney and body weight and is often used to assess the renal health condition.

### 2.5 Evaluation of renal function

Fresh blood samples were centrifuged at 3000 rpm for 15 min at 4 °C to obtain serum. The serum levels of creatinine (CREA) and uric acid (UA) were measured using a fully automated biochemical analyzer (BS-240, Mindray Bio-Medical Electronics Co., Ltd., Shenzhen, China).

### 2.6 Hematoxylin–eosin staining (HE staining)

Fresh kidney tissues were fixed in 4% paraformaldehyde for 24 h, followed by dehydration in a graded ethanol series, clearing with xylene, and paraffin embedding. The wax blocks were cut into 5 μm-thick sections and stained with hematoxylin–eosin (HE) solution according to a previously described method (23). Finally, the sections were observed and photographed using an optical microscope (Olympus, Tokyo, Japan).

### 2.7 Ultrastructure observation

A 1-mm^3^ piece of duck kidney tissue was cut and fixed in 2.5% glutaraldehyde and 1% osmium acid solutions for 12 h and 2 h, respectively. Then, the tissue was dehydrated using a graded ethanol series and immersed in a 1:1 mixture of epoxy resin and acrylic for 2 h. Next, the sample was embedded in epoxy resin. After high-temperature baking, the sample was sectioned using an ultrathin sectioning machine, and the sections were placed flat on a copper plate. After drying, they were stained successively with 2% acetic anhydride, uranium, and lead citrate. After washing off the residual liquid with ultrapure water, the stained copper plate was placed on a filter paper for observation. The changes were then examined and captured using a transmission electron microscope (JEM1400, Tokyo, Japan).

### 2.8 Detection of TC and TG levels

According to the manufacturer’s instructions, the TC and TG contents in serum and kidney tissue were determined (Jiancheng Biotech, Nanjing, China).

### 2.9 Ion content detection

After drying the duck kidney at 65 °C for 24 h, the tissue was ground into powder, weighed, and recorded. Then, hydrogen peroxide and nitric acid were mixed at a ratio of 1:5, and the tissue powder was digested using a microwave system. The digestion solution was diluted with deionized water, and the levels of manganese (Mn), Cd, zinc (Zn), iron (Fe), and copper (Cu) in the kidney were detected using flame atomic absorption spectrometry (Optima 3700DV, PerkinElmer, USA).

### 2.10 Determination of oxidative stress indicators

To determine the levels of glutathione (GSH), malondialdehyde (MDA), and hydrogen peroxide (H₂O₂), as well as the activities of total antioxidant capacity (T-AOC), catalase (CAT), and superoxide dismutase (SOD), the kidney sample and saline at a ratio of 1: 9 (w/v) were mixed to prepare the homogenate. The homogenate was then centrifuged at 3000 g for 10 min at 4 °C. Subsequently, the relevant indicators were determined using kits (Jiancheng Biotech, Nanjing, China).

### 2.11 Enzyme-linked immunosorbent assay (ELISA)

After homogenizing the kidney sample with an appropriate volume of physiological saline, the homogenate was centrifuged at 3000 rpm for 10 min at 4 °C to detect the levels of the inflammatory cytokines tumor necrosis factor-α (TNF-α), interleukin-18 (IL-18), and interleukin-1β (IL-1β) (Mlbio, Shanghai, China).

### 2.12 Immunofluorescence staining and analysis

Paraffin sections were first subjected to dewaxing and dehydration treatment, and then antigen remediation was carried out using microwave heating with sodium citrate buffer solution. Next, 0.5% Triton X-100 and 5% bovine serum albumin were used for membrane permeation and blocking, respectively. Subsequently, the primary antibody was incubated overnight at 4 °C in a wet box. The fluorescence secondary antibody was incubated at room temperature for 2 h. After washing three times with PBS, the plate was sealed with an anti-fluorescence quencher containing DAPI. Finally, the sections were observed and photographed using a fluorescence microscope (Leica Microsystems, Nussloch, Germany). Visual analysis of fluorescence intensity was performed using the ImageJ software.

### 2.13 Western blotting

The kidney sample was lysed in RIPA buffer supplemented with PMSF, and the lysate was centrifuged at 12,000 rpm for 15 min at 4 °C. The resulting supernatant was collected. Next, the BCA method was used to detect and quantify the protein concentration. Proteins of different molecular weights were separated by SDS-PAGE and transferred onto the NC membrane. After incubating with 5% skimmed milk for 2 h, the membrane was successively incubated with the primary antibody at 4 °C overnight and with the secondary antibody at room temperature for 2 h. Finally, the membranes were washed three times with TBST, and protein signals were observed and quantified using the ECL kit and ImageJ software (Beyotime, Shanghai, China). All the antibodies used in this study are shown in Table 1.

**Table 1:** Antibodies used in the present study.

Antibody	Article number	Company
NRF2	T55136F	Abmart
HO-1	TA5393	Abmart
NQO1	TD6437	Abmart
GCLC	A1038	ABclonal
GCLM	A5314	ABclonal
GSR	A4566	ABclonal
FAS	A19582	ABclonal
SREBP1	A26708	Cell signaling technology
ACC1	A0994	ABclonal
ACC2	A0134	ABclonal
PPARα	A6697	ABclonal
ATGL	A5126	ABclonal
HSL	A15686	ABclonal
PPARγ	A0270	ABclonal
CPT2	A12426	ABclonal
LPL	T40056	Abmart
iNOS	A3774	ABclonal
CD68	A6554	ABclonal
Arg1	A1847	ABclonal
CD4	A0362	ABclonal
GATA3	A19636	ABclonal
T-bet	A25647	ABclonal
β-actin	4,967 L	Cell signaling technology
Against rabbit IgG	3900S	Cell signaling technology
Against mouse IgG	4416S	Cell signaling technology

### 2.14 Statistical analysis

All experimental data are presented as mean ± standard deviation (SD) and were statistically analyzed using SPSS 25.0 (IBM, Armonk, NY, USA). Differences between groups were analyzed using Fisher’s least significant difference (LSD) and one-way analysis of variance (ANOVA), and the chart was generated using GraphPad Prism 9.0 (GraphPad Software, San Diego, CA, USA) and ImageJ 1.8.0 (National Institutes of Health, Bethesda, MD, USA). A *p*-value of > 0.05 indicated no statistical significance, while a *p*-value of < 0.05 indicated statistical significance.

## 3 Results

### 3.1 LBP ameliorates cd-induced changes in the kidney index and kidney function in ducks

The body weight of the ducks was significantly lower (*p* < 0.001), while kidney weight and the kidney index were significantly higher (*p* < 0.01 or *p* < 0.001) in the Cd group compared to the control group. Conversely, body weight was significantly higher (*p* < 0.05), while kidney weight and the kidney index were significantly lower (*p* < 0.05) in the Cd + LBP group compared to the Cd group (Figures 1B–D). Moreover, Cd increased the levels of the renal function indicators CREA and UA, whereas LBP supplementation markedly reduced (*p* < 0.001) the levels of both indicators (Figures 1E,F). These results show that Cd disrupted kidney function and induced nephrotoxicity, while LBP improved Cd-induced nephrotoxicity.

### 3.2 LBP improves cd-induced histopathological damage in duck kidneys

HE staining is a commonly used method for observing and assessing the extent of tissue injury. As depicted in Figure 2A, the kidneys in the control group appeared normal, with no obvious pathological changes. Compared to the control group, the renal structure was severely damaged, with indistinct boundaries, atrophied and deformed glomeruli, and severe interstitial hemorrhage in the Cd group. Compared to the Cd group, LBP and Cd co-treatment markedly alleviated Cd-induced structural damage and improved inflammatory cell infiltration. Furthermore, the results of renal microenvironment observations are shown in Figure 2B. The microenvironment structure was abnormal in the treatment group, with nuclear deformation, severe mitochondrial vacuolation, and mitochondrial cristae rupture. However, this poor condition caused by Cd exposure can be effectively improved by administering LBP additives. These results suggest that Cd induced histological and ultrastructural damage to the kidneys, while LBP can effectively ameliorate Cd-induced kidney damage.

**Figure 2:**
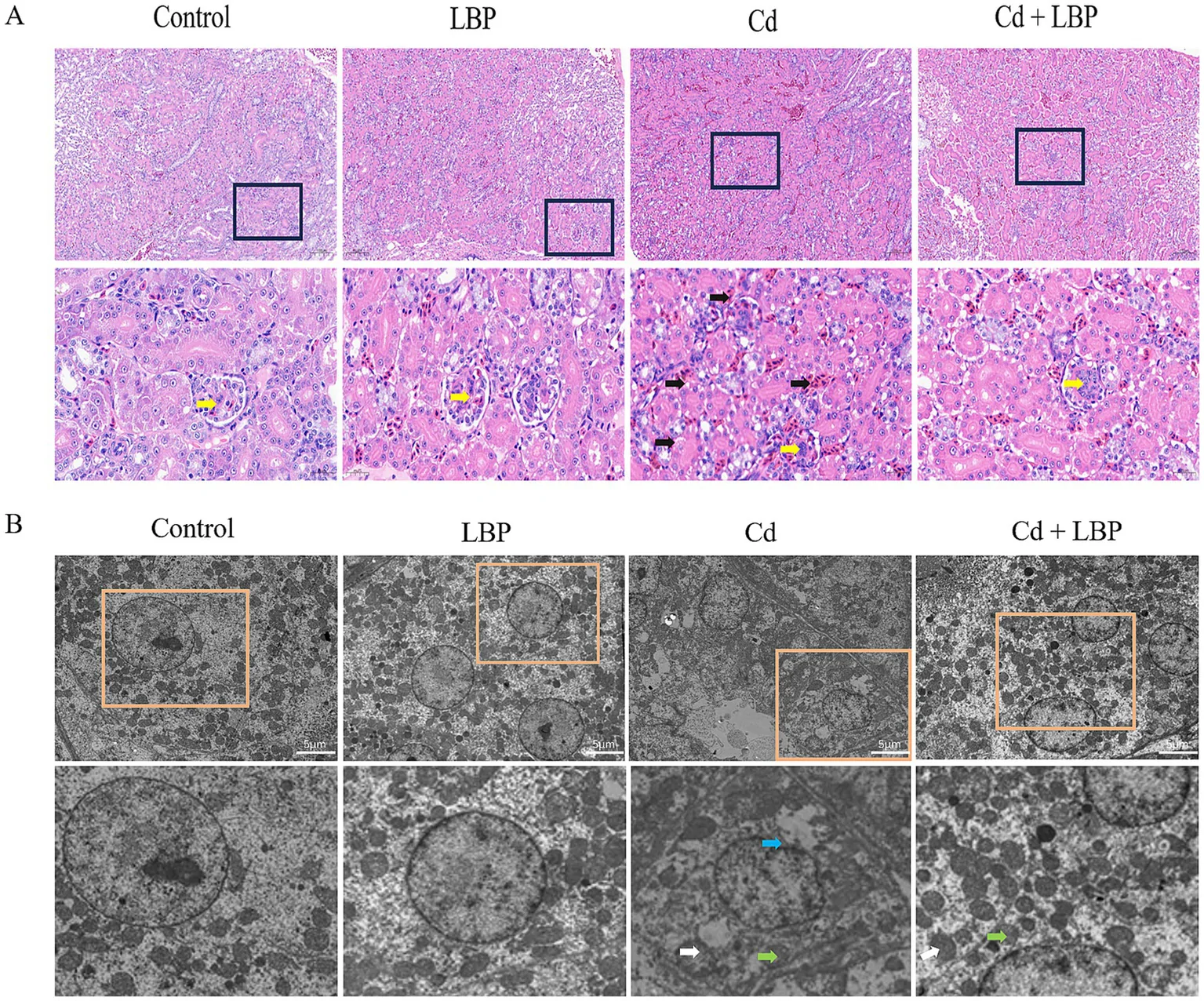
LBP improved pathological injury and ultrastructural changes in the Cd-exposed duck kidneys. **(A)** HE staining. Yellow arrow: glomerulus. Black arrow: interstitial hemorrhage. Scale bar: 100 μM. **(B)** Ultrastructure observation. Blue arrow: nuclear deformation. White arrow: mitochondrial vacuolation. Green arrow: mitochondrial cristae rupture. Scale bar: 5 μM.

### 3.3 LBP restores cd-disrupted ion homeostasis in duck kidneys

Trace elements participate in the composition of various oxidant and antioxidant enzymes and are closely linked to the occurrence and development of oxidative stress. We measured the renal ion levels, and the results are presented in Figures 3A–E. Compared to the control group, Cd exposure increased Cd content while reducing the levels of Fe, Mn, Zn, and Cu. Interestingly, LBP and Cd co-exposure decreased Cd content while increasing the levels of Fe, Mn, Zn, and Cu compared to the Cd group. The trend of ion content changes is more intuitively illustrated by the heatmap (Figure 3F). These results report that LBP alleviated Cd-induced disruption of ion homeostasis in the duck kidney.

**Figure 3:**
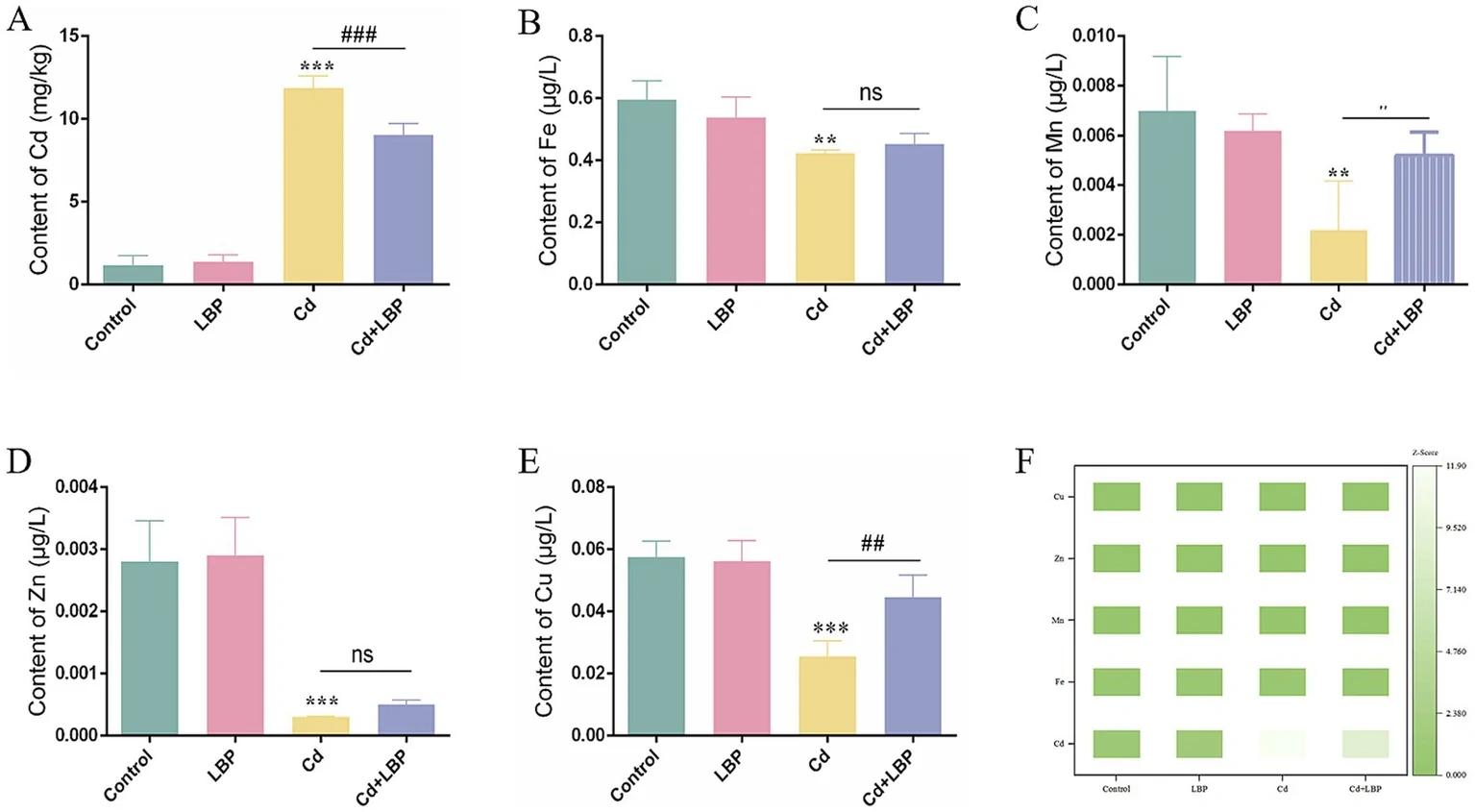
LBP alleviated kidney injury in Cd-exposed ducks. **(A)** Experimental design of the study. **(B)** Body weight. **(C)** Kidney weight. **(D)** Kidney index. **(E)** Serum CREA. **(F)** Serum UA. All results are presented as the mean ± SD. *p < 0.05, **p < 0.01, and ***p < 0.001 compared to the control group; #*p* < 0.05, ##*p* < 0.01, and ###*p* < 0.001 compared to the Cd group; ns represents *p* > 0.05.

### 3.4 LBP modulates the Nrf2-mediated antioxidant defense pathway to counteract cd-induced oxidative stress

To explore the impact of Cd on oxidative stress, we examined several oxidative stress-related indexes. As shown in Figures 4A–F, MDA and H_2_O_2_ contents were markedly elevated, while the activities of SOD, T-AOC, and CAT were markedly decreased in the Cd group compared to the control group. However, the above changes were reversed following LBP addition. GSH content was markedly lower in the Cd group than in the control group, but it was significantly increased in the LBP and Cd co-exposure group compared to the Cd group. The radar chart more clearly shows the changes in these indicators for each group (Figure 4G). For the Nrf2-mediated antioxidant signaling axis, we measured the levels of pathway-related proteins. Figures 5A–G demonstrates that the Cd group had evidently lower protein levels of Nrf2, NQO1, HO-1, GCLM, GCLC, and GSR compared to the control group. However, these protein expression levels in the Cd + LBP group were substantially higher compared to the Cd group. The heatmap facilitates an intuitive understanding of the changes in protein expression levels across the different groups (Figure 5H). These findings indicate that LBP ameliorated Cd-induced oxidative stress by activating the Nrf2-mediated antioxidant signaling axis.

**Figure 4:**
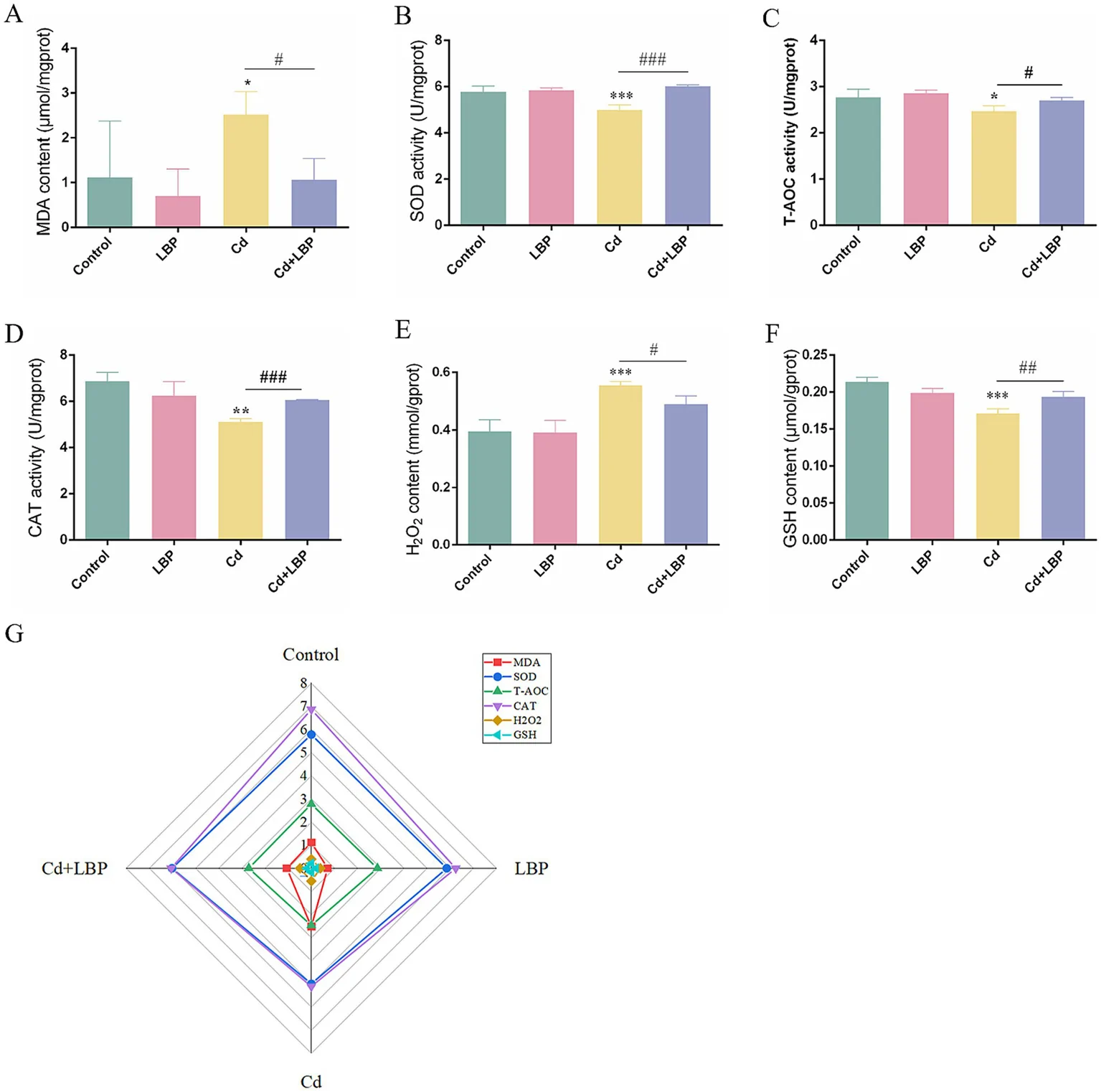
LBP inhibited renal oxidative stress induced by Cd. **(A)** MDA content. **(B)** SOD activity. **(C)** T-AOC activity. **(D)** CAT activity. **(E)** H_2_O_2_ content. **(F)** GSH content. **(G)** Radar chart. All results are presented as the mean ± SD. *p < 0.05, **p < 0.01, and ***p < 0.001 compared to the control group; ^#^*p* < 0.05, ^##^*p* < 0.01, and ^###^*p* < 0.001 compared to the Cd group; ns represents *p* > 0.05.

**Figure 5:**
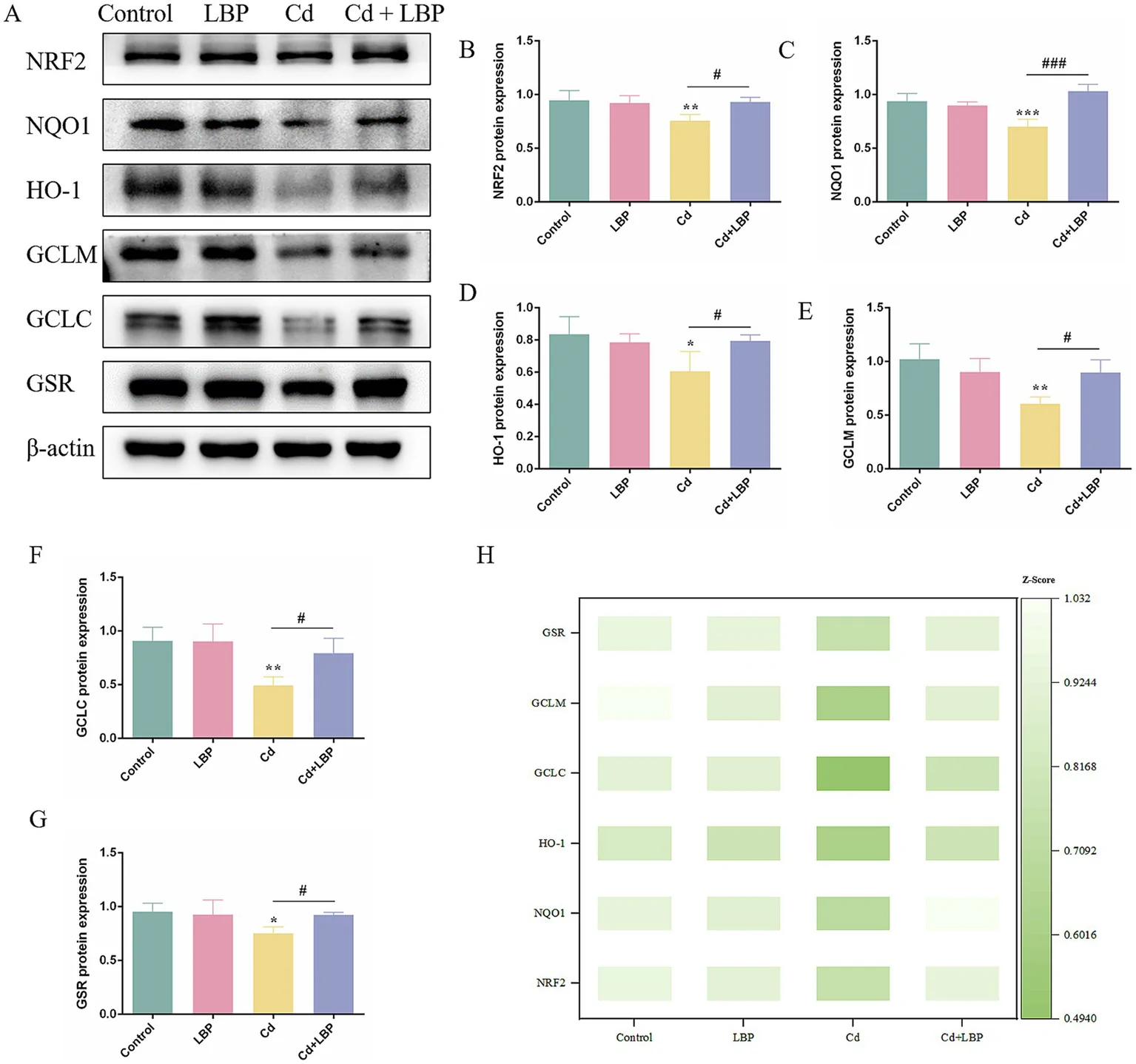
LBP activated the NRF2-mediated antioxidant defense pathway inhibited by Cd exposure. **(A)** Protein bands. **(B)** NRF2 expression level. **(C)** NQO1 expression level. **(D)** HO-1 expression level. **(E)** GCLM expression level. **(F)** GCLC expression level. **(G)** GSR expression level. **(H)** Heatmap analysis. The heatmap color gradient shows row-wise z-score-normalized protein levels (positive = higher relative content and negative = lower relative content). Darker colors indicate lower expression levels, while lighter colors represent higher expression levels. Abbreviations: NRF2, nuclear factor erythroid 2-related factor 2; NQO1, NAD(P)H quinone dehydrogenase 1; HO-1, heme oxygenase-1; GCLM, glutamate-cysteine ligase modifier subunit; GCLC, glutamate-cysteine ligase catalytic subunit; GSR, glutathione reductase. All results are presented as the mean ± SD. *p < 0.05, **p < 0.01, and ***p < 0.001 compared to the control group; ^#^*p* < 0.05, ^##^*p* < 0.01, and ^###^*p* < 0.001 compared to the Cd group; ns represents *p* > 0.05.

### 3.5 LBP attenuates cd-induced lipid metabolism disorder in duck kidneys

TEM analysis revealed that LBP reduced the accumulation of lipid droplets induced by Cd (Figure 6E). Consistent with these findings, compared to the control group, TC and TG levels were evidently elevated in the Cd group. In contrast, compared to the Cd group, TC and TG levels were significantly decreased in the serum and kidney following co-exposure to Cd and LBP (Figures 6A–D). The expression levels of lipid metabolism-related proteins FAS, SREBP1, ACC1, ACC2, and PPARγ were markedly upregulated after Cd treatment compared to the control group. However, these protein expression levels were significantly downregulated after LBP and Cd co-exposure (Figures 6F–K). Conversely, compared to the control group, Cd exposure downregulated the expression levels of ATGL, HSL, PPAR α, CPT2, and LPL, whereas the expression levels of these proteins were markedly upregulated after LBP and Cd co-exposure (Figures 6L–Q). The results show that LBP alleviated lipid accumulation induced by Cd.

**Figure 6:**
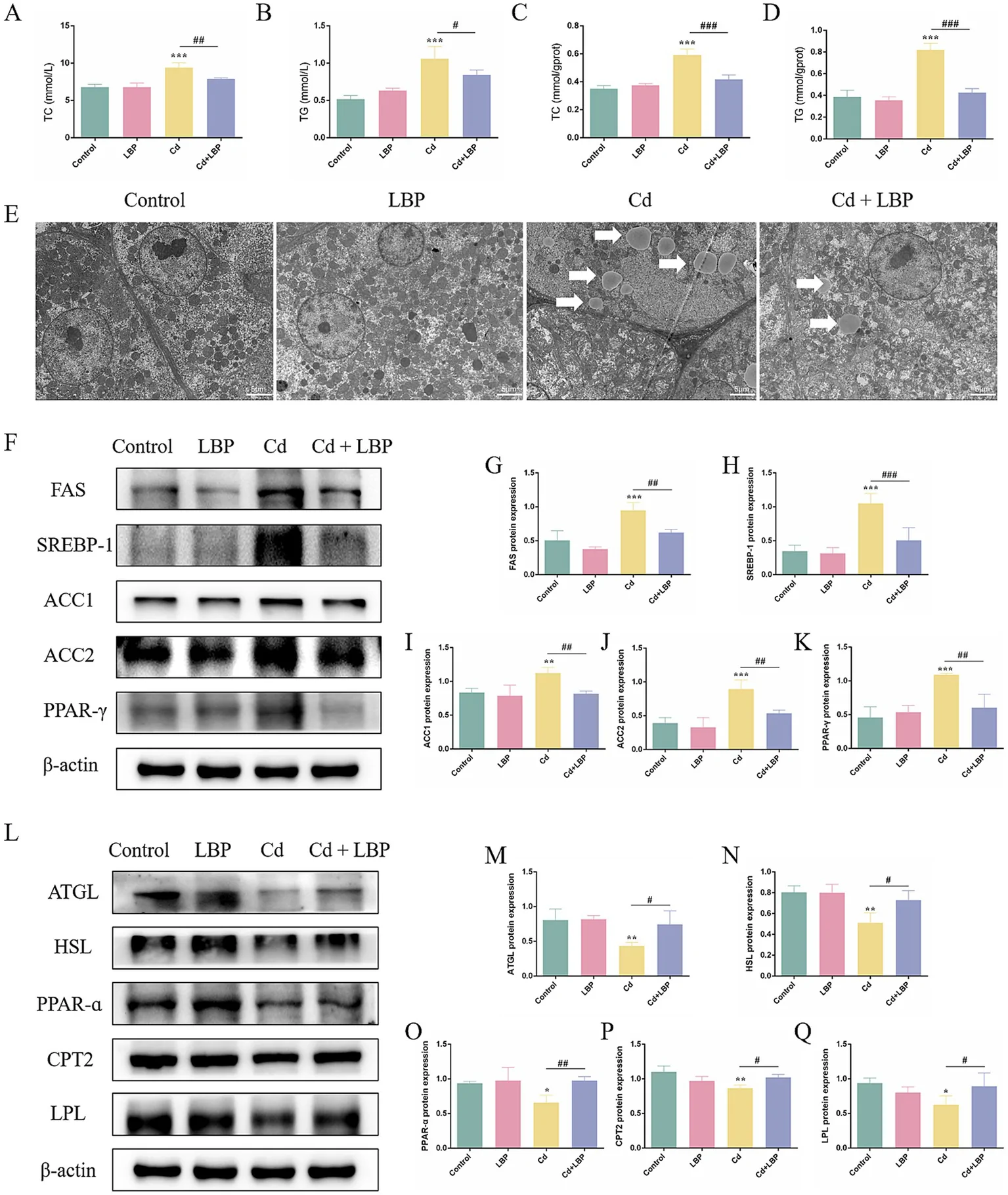
LBP effectively alleviated renal lipid metabolism disorder in ducks under Cd stress. **(A)** Serum TC level. **(B)** Serum TG level. **(C)** Renal TC level. **(D)** Renal TG level. **(E)** Observation of lipid droplets. Scale bar: 5 μM. **(F)** Protein bands. **(G)** FAS expression level. **(H)** SREBP-1 expression level. **(I)** ACC1 expression level. **(J)** ACC2 expression level. **(K)** PPAR-γ expression level. **(L)** Protein bands. **(M)** ATGL expression level. **(N)** HSL expression level. **(O)** PPAR-α expression level. **(P)** CPT2 expression level. **(Q)** LPL expression level. All results are presented as the mean ± SD. *p < 0.05, **p < 0.01, and ***p < 0.001 compared to the control group; ^#^*p* < 0.05, ^##^*p* < 0.01, and ^###^*p* < 0.001 compared to the Cd group; ns represents *p* > 0.05.

### 3.6 LBP alleviates the adverse effects of Cd on immune homeostasis in duck kidneys

To determine whether Cd exposure and renal immunity were related, we examined the expression levels of immune-related factors. The results of the immunofluorescence analysis are shown in Figures 7A,B. The fluorescent signal of iNOS was increased, while the fluorescent signal of Arg-1 was decreased in the Cd group compared to the control group. Conversely, the fluorescent signal of iNOS was decreased, while the fluorescent signal of Arg-1 was elevated in the Cd + LBP group compared to the Cd group. Similarly, the expression levels of iNOS, CD68, T-bet, and CD4 were significantly upregulated in the Cd group compared to the control group, whereas the expression levels of Arg1 and GATA3 were significantly downregulated. In addition, the expression levels of iNOS, CD68, T-bet, and CD4 were markedly downregulated in the Cd + LBP group compared to the Cd group, while the expression levels of Arg1 and GATA3 were significantly upregulated (Figures 7C–I,M). Furthermore, compared to the control group, Cd increased the levels of the inflammatory factors IL-18, IL-1B, and TNF-α. Compared to the Cd group, the levels of these inflammatory factors were markedly reduced in the Cd and LBP combined group (Figures 7J–L,N). The results suggest that LBP attenuated Cd-induced immunosuppression and inflammatory responses.

**Figure 7:**
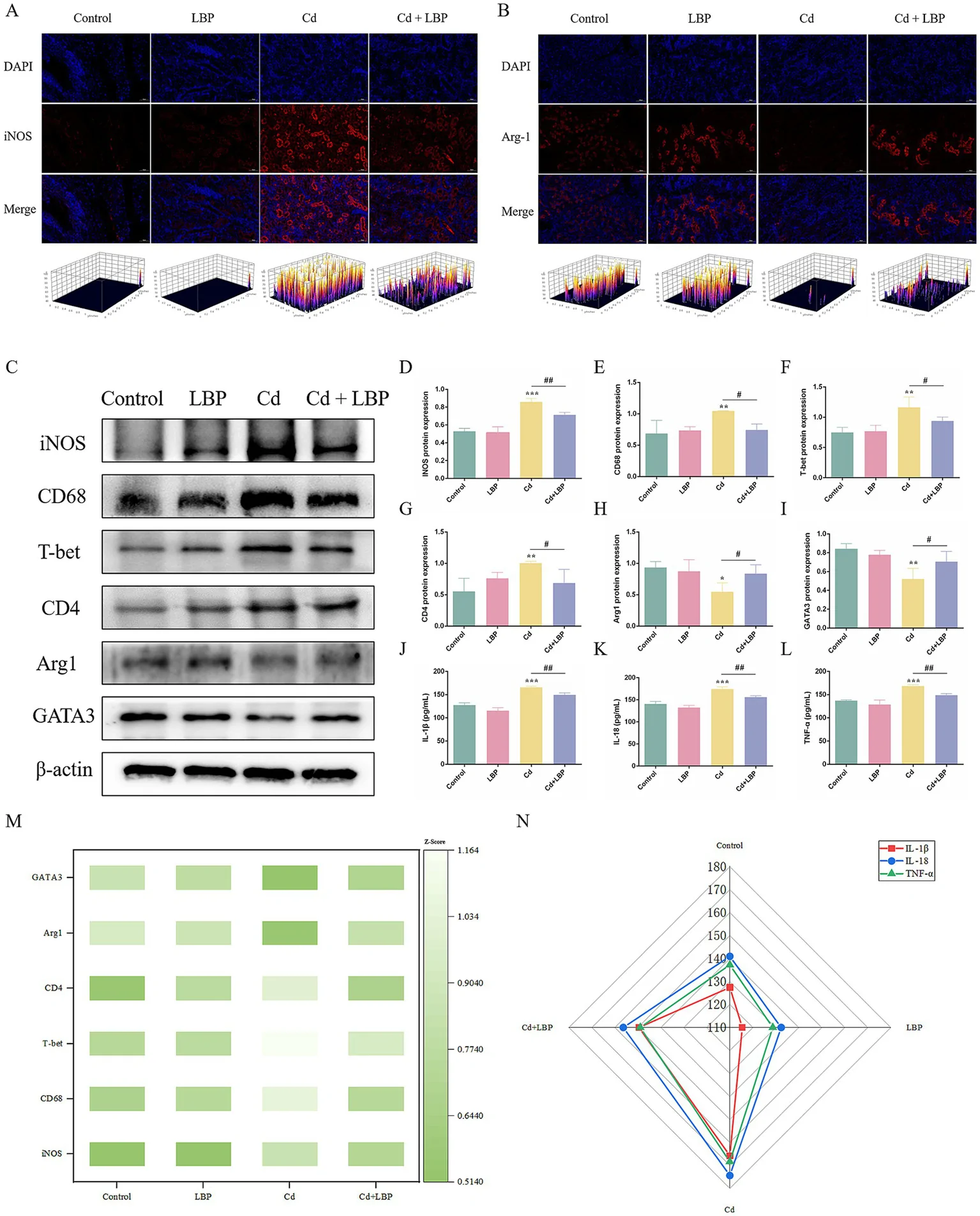
LBP restored immune balance and inhibited Cd-induced kidney inflammation. **(A)** Immunofluorescence observation of iNOS. **(B)** Immunofluorescence observation of Arg1. **(C)** Protein bands. **(D)** iNOS expression level. **(E)** CD68 expression level. **(F)** T-bet expression level. **(G)** CD4 expression level. **(H)** Arg1 expression level. **(I)** GATA3 expression level. **(J)** IL-1β content. **(K)** IL-18 content. **(L)** TNF-α content. **(M)** Heatmap analysis. **(N)** Radar chart. All results are presented as the mean ± SD. *p < 0.05, **p < 0.01, and ***p < 0.001 compared to the control group; ^#^*p* < 0.05, ^##^*p* < 0.01, and ^###^*p* < 0.001 compared to the Cd group; ns represents *p* > 0.05.

## 4 Discussion

In the current study, we explored the protective effects of LBP through Nrf2- mediated antioxidant activity in the kidneys of Cd-exposed ducks. The results confirmed a remarkable therapeutic effect of LBP in alleviating Cd-induced oxidative damage, immune imbalance, and lipid metabolism disorder by enhancing renal antioxidant function via the Nrf2 signaling pathway. The mechanism of action was achieved through the antioxidant, anti-inflammatory, and lipid-lowering effects of LBP itself. This study not only provides a novel scientific perspective for clarifying the traditional efficacy of *Lycium barbarum* but also offers molecular evidence for its clinical significance in preventing or treating Cd-induced immune suppression and lipid metabolism disorder.

Cd is a non-essential element for vertebrates. However, as a pollutant with strong accumulation capacity, it has long been a hot topic in global toxicology investigation. As early as 1992, the ATSDR ranked Cd as the sixth most harmful toxic substance to human health. Subsequently, the IARC classified Cd as a Class A carcinogen in 2018 (24). Currently, with the continuous development of domestic industrialization, a large amount of heavy metal-containing waste is discharged, leading to serious environmental pollution problems and posing a serious threat to human health (25). Previous research has revealed that Cd decreases the growth rate and feed intake while increasing the levels of the liver function indicators ALT, AST, and ALP in ducks (22). Similarly, in this study, we found a significant reduction in body weight and marked increases in kidney weight, the kidney index, and the kidney function indicators CREA and UA in Cd-exposed ducks. Moreover, Cd caused histopathological and ultrastructural damage to the kidney, which is consistent with the investigation of Zhang et al. (26). A previous study showed that Cd exposure promoted Cd accumulation in the kidney and disrupted trace element homeostasis, leading to tissue damage and inflammatory responses (27). Maintaining trace element homeostasis is important for the normal growth, development, and physiological activities of animals. Heavy metal exposure can promote their accumulation and competitive binding with corresponding metal ions in the body, thereby disrupting trace element homeostasis (28). In this study, Cd accumulation disrupted the homeostasis of other trace elements and triggered oxidative stress, as evidenced by increased MDA and H_2_O_2_ levels and decreased SOD, T-AOC, and CAT activities and GSH content. When the production of free radicals exceeds the clearance capacity of the antioxidant system in the body, the balance is disrupted. Excessive free radicals will attack cells, causing oxidative damage to the cells. Trace elements are essential components of key antioxidant enzymes in the body. Once lacking, the corresponding enzyme activity will decline, and the body’s ability to eliminate free radicals will weaken, thereby causing oxidative stress (29). For instance, Se is the core component of glutathione peroxidase (GPx). This enzyme is one of the most important antioxidant enzymes in the body and is responsible for breaking down hydrogen peroxide (H₂O₂) and lipid peroxides, thereby protecting cell membranes from oxidative damage (30). Se deficiency can directly lead to a significant reduction in GPx activity, causing the accumulation of harmful peroxides and triggering severe lipid peroxidation and cell membrane damage. Zn is a component of superoxide dismutase (SOD), an enzyme that is responsible for converting the most toxic superoxide anion (O₂^−^) into hydrogen peroxide (H₂O₂) (31). Zn deficiency leads to a decrease in SOD activity and causes more iron to participate in the Fenton reaction, thereby intensifying oxidative stress. Cu is an essential component of another superoxide dismutase (CuZn-SOD), working in synergy with Zn. Cu deficiency also reduces SOD activity and its ability to remove superoxide anions. Mn is a cofactor of manganese superoxide dismutase (Mn-SOD) in mitochondria and is mainly responsible for eliminating superoxide anions produced by mitochondria (32). Mn deficiency can particularly affect the antioxidant defense of mitochondria, causing damage to energy production. Excessive free iron (especially Fe^2+^) can catalyze hydrogen peroxide (H₂O₂) to generate highly destructive hydroxyl radicals (OH) through the Fenton reaction. The fundamental cause of this phenomenon is that Cd is a toxic heavy metal. It exhibits chemical and biological properties similar to those of zinc, iron, manganese, and copper (such as ionic radius and coordination tendency), which enables it to “impersonate” these essential elements in the body, but it fails to perform their physiological functions, thereby interfering with and disrupting normal physiological and biochemical processes (33). Importantly, we found that the natural plant component LBP extracted from wolfberry significantly improved Cd-induced renal pathological damage and oxidative stress through its own antioxidant effect. This finding was further confirmed in studies conducted by Wei et al. (34) and Ren et al. (35).

Oxidative stress is a major contributor to Cd-induced nephrotoxicity. Long-term exposure to Cd elevates reactive oxygen levels while also reducing the body’s antioxidant capacity. The kidney is a primary target organ of Cd toxicity and is particularly vulnerable to damage from oxidative stress. Nrf2 is a major transcription factor that promotes the transcription of antioxidant response element (ARE)-dependent genes. Its antioxidant defense response plays a crucial role in combating oxidative damage caused by toxic substances (36). Normally, when cells are exposed to adverse stimuli, the stability of Keap1-Nrf2 binding is disrupted and activated Nrf2 is quickly translocated into the nucleus to bind to the ARE, thereby activating the transcription of downstream-related factors HO-1 and NQO1 to protect cells from injury (37). Sprague–Dawley rats treated with increasing amounts of Cd showed evidently decreased Nrf2 and GCLM protein expression, elevated ROS accumulation, and markedly reduced antioxidant enzyme activity (38). Relevant research has demonstrated that Cd exerts toxic effects by downregulating and/or upregulating the expression of Nrf2 and Keap1, which triggers the cytoplasmic degradation of their downstream proteins HO1 and NQO1, thereby suppressing antioxidant capacity (39). Our findings show that Cd not only inhibited the expression of Nrf2 and HO-1 but also reduced the expression of NQO1, GCLC, GCLM, and GSR, thereby causing oxidative stress. After LBP treatment, we observed that the dysregulated expression of proteins associated with the Nrf2-mediated antioxidant signaling axis was improved, indicating that LBP alleviates Cd-induced oxidative stress. The core components of the glutathione synthesis and regeneration system include GCLC, GCLM, and GSR, and all of them are directly regulated by the NRF2 signaling pathway (40). GCLC is a heterodimeric protein, and it is also the rate-limiting enzyme in the process of GSH synthesis, responsible for regulating the first step of glutathione synthesis. GCLM itself does not possess catalytic activity. When it combines with GCLC, it forms a complete whole-enzyme GCL, promoting the generation of a large amount of GSH. GSR catalyzes the reduction of oxidized glutathione (GSSG) to reduced glutathione (GSH), a critical step for glutathione regeneration within the glutathione cycle. When the Nrf2 pathway is activated, the expression levels of GCLC, GCLM, and GSR are also upregulated. This not only means that cells accelerate the production of new glutathione but also that they enhance the ability to recycle old glutathione (41). Therefore, the downstream core components of the Nrf2 pathway with antioxidant functions jointly maintain the homeostasis of glutathione, the most important cellular antioxidant. Interestingly, we found that Cd reduced the synthesis of GSH by inhibiting the levels of GCLC, GCLM, and GSR, thereby causing oxidative stress, which, to some extent, explains the specific mechanism of the Nrf2 pathway antioxidant effect. Sun et al. also confirmed that Cd-induced oxidative stress is achieved by reducing glutathione peroxidase (GSH-Px) and total superoxide dismutase (T-SOD) activities, increasing the concentration of malondialdehyde (MDA), and downregulating the transcriptional levels of antioxidant genes (GCLC, GCLM, and GSR) in the duck liver (42). This is significantly consistent with our research results.

In recent years, numerous studies have reported that lipid metabolism disorder is a core link in Cd-induced nephrotoxicity. Studies based on clinical serum samples have shown that Cd exposure alters the levels of phosphatidylcholine (PC), triglycerides (TG), cholesterol esters (CE), lysophosphatidylcholine (LPC), and ceramides (Cer) (43). Coincidentally, these findings were verified in the study conducted by Gu et al. (44) Long-term exposure to low doses of Cd impairs the intestinal microbiota and lipid metabolism profile, thereby causing liver damage. Lipid metabolism disorder, also known as dyslipidemia, refers to any abnormality in the process of lipid synthesis, decomposition, transportation, or storage within the body that leads to an imbalance in blood lipid levels or abnormal lipid accumulation in non-fat tissues. Zhang et al. (45) reported that Cd disrupted the lipid metabolism balance in a mouse model, resulting in lipid accumulation in the liver. In Cd-exposed rats, Oluranti et al. noted that, compared to the normal group, the levels of free fatty acids, total cholesterol, and triglycerides were increased significantly with increasing Cd doses, leading to excessive lipid accumulation and thereby impairing cardiac function (46). Similarly, in the present study, we observed that Cd elevated TC and TG levels in the serum and kidneys. Moreover, a large number of lipid droplets accumulated in the kidneys. ATGL is a rate-limiting enzyme in the fat breakdown process responsible for catalyzing the first step of triglyceride decomposition, hydrolyzing it into diglycerol and a free fatty acid. HSL mainly hydrolyzes diglycerol to produce monoglyceride and another free fatty acid. Paternal Cd exposure promoted LD accumulation in ovarian tissue by inhibiting the expression of the LD degradation-related proteins ATGL and HSL in offspring (47). PPARα is the main regulatory transcription factor of fatty acid oxidation. Once activated, it can upregulate the expression of a series of fatty acid oxidation-related factors, such as CPT1 and CPT2. CPT2 is the final step in the process through which long-chain fatty acids enter the mitochondrial matrix for β-oxidation. Both *in vivo* and *in vitro*, CPT1 deficiency promotes Cd-induced lipid accumulation, mainly presented as an increase in protein ACC expression and a decrease in CPT1 and PPARα expression (48). FAS is the central executor in fatty acid synthesis. SREBP-1 is the main regulatory transcription factor for fatty acid and triglyceride synthesis. After activation, it can upregulate the expression of all genes required for fatty acid synthesis, such as the key enzymes ACC and FAS. CdCl_2_ upregulates the expression of PPARγ, FAS, and SREBP1 in the muscle of tilapia (49). PPAR-γ is a key factor in adipocyte differentiation and lipid storage. After activation, it promotes the maturation of adipocytes and upregulates the expression of genes involved in lipid uptake (such as LPL) and storage (such as FAS and SREBP-1). LPL is a rate-limiting enzyme in the process of energy metabolism. Lipoprotein lipase (LPL) catalyzes the breakdown of circulating triglyceride rich lipoproteins, hydrolyzing them into free fatty acids and glycerol. Hirano et al. reported elevated triglyceride levels concurrent with reduced tissue LPL activity in CdŁexposed mice (50). In the present study, Cd altered the expression of these key proteins to promote lipogenesis, which disturbs lipid metabolism and triggers aberrant lipid accumulation in the kidney, eventually leading to lipotoxicity and renal injury.

Long-term oxidative stress and inflammatory responses can interfere with lipid metabolism and cause abnormal lipid deposition. This metabolic disorder further aggravates inflammation, impairing the immune system and forming a vicious cycle. Lipid reprogramming not only maintains cellular energy homeostasis but also acts as an essential modulator of immune cell phenotypes. Persistent inflammation also causes immune cell fatigue, thereby triggering immunosuppression (51). The immune system is an extremely important defense barrier of the organism (52). It is highly sensitive to external pollutants and can promptly eliminate and recognize non-internal macromolecular substances, maintaining internal and external balance. It is composed of immune cells, immune organs, and immune active substances. The kidney is an organ with immunomodulatory functions, mainly containing lymphocytes and macrophages and maintaining the stability of the internal immune environment by eliminating metabolic waste. The health of the body is closely linked to the normal immune system. Research has found that exposure to heavy metals not only causes toxic effects in the human body but also leads to immunosuppression. For instance, Cd bioaccumulation impairs the immune system by reducing the number of immune cells and their phagocytic activity, potentially disrupting immune balance and weakening immunosurveillance (53). The immune response is achieved by immune cells releasing cytokines. Among them, macrophages can differentiate into two distinct subtypes: Classically activated M1 macrophages and alternatively activated M2 macrophages. M1 macrophages are polarized into cells that secrete pro-inflammatory factors (such as IL-12), thereby accelerating the inflammatory response. M2 macrophages produce anti-inflammatory factors, such as IL-10 and TGF-β, which can inhibit inflammation and restore damaged tissues. A report on subacute Cd exposure found that Cd induced oxidative stress, caused inflammation, and accelerated the polarization of M1-type macrophages, ultimately causing adrenal fibrosis in pigs (54). Furthermore, the Th1 and Th2 lymphocyte subtypes play similar roles in regulating inflammatory reactions and immune functions in the organism. T1 and T2 cells, respectively, produce pro-inflammatory factors and anti-inflammatory factors. *In vivo* experiments showed that PBQ caused immune dysfunction by disrupting the Th1/Th2 cytokine balance and the apoptotic pathway in the mouse spleen (55). In a mouse model of atherosclerosis, it was found that the ratio of macrophages and lymphocytes was imbalanced, as evidenced by elevated protein expression levels of iNOS and T-bet and decreased protein expression levels of Arg-1 and GATA-3 (56). In the current study, we also examined the levels of polarization-related markers in macrophages and lymphocytes. Cd exposure upregulated the levels of the macrophage markers iNOS and CD68, as well as the expression levels of the lymphocyte markers T-bet and CD4, while downregulating the expression levels of Arg1 and GATA3. This finding is consistent with that reported by Wang et al. (57). Furthermore, LBP supplementation can effectively alleviate the immune imbalance caused by Cd. The above research results have confirmed that LBP indeed can enhance immune function and suppress inflammatory responses.

## 5 Conclusion

In conclusion, our findings suggest that Cd induces oxidative stress and inflammatory reactions by disrupting trace element homeostasis and promotes immune imbalance by disrupting lipid metabolism. In addition, LBP supplementation can effectively alleviate the abovementioned damage, indicating that LBP is a potential drug that could be used to treat renal toxicity caused by Cd.

## 6 Novelty and limitations

Although prior poultry studies have reported that LBP alleviates heavy metal-induced oxidative injury via Nrf2 signaling, the novelty of the present study lies in its comprehensive characterization of disrupted trace element homeostasis, lipid metabolism reprogramming, and M1/M2-Th1/Th2 immune imbalance in Cd-damaged duck kidneys, extending knowledge beyond the classic Nrf2 antioxidant response. Nevertheless, this study has limitations. All findings are derived from phenotypic observations and targeted protein measurements in an *in vivo* duck model. In the absence of Nrf2 inhibitor or gene-interference assays, the observed Nrf2 changes only indicate correlation rather than causality. Moreover, transcriptomic and metabolomic analyses were not performed, restricting the exploration of global molecular alterations. Untargeted omics analyses and functional validation will be conducted in future research to discover additional targets.

## Data Availability

The original contributions presented in the study are included in the article/supplementary material, further inquiries can be directed to the corresponding authors.
